# Ratooning Annual Cotton (*Gossypium* spp.) for Perennial Utilization of Heterosis

**DOI:** 10.3389/fpls.2020.554970

**Published:** 2020-12-03

**Authors:** Xin Zhang, Zhiyong Zhang, Ruiyang Zhou, Qinglian Wang, Linsong Wang

**Affiliations:** ^1^Biological Postdoctoral Research Station, Henan Normal University, Xinxiang, China; ^2^Postdoctoral Research Base, Henan Collaborative Innovation Center of Modern Biological Breeding, Henan Institute of Science and Technology, Xinxiang, China; ^3^College of Agronomy, Guangxi University, Nanning, China

**Keywords:** stub, tropics, hybrid, seed production, male sterile, asexual reproduction, cutting, grafting

## Abstract

This paper reviews an important topic within the broader framework of the use of ratoon cotton for the development of a cost-saving and efficient method for the perennial production of hybrid cotton seeds. Cotton has a botanically indeterminate perennial growth habit and originated in the tropics. However, cotton has been domesticated as an annual crop in temperate areas worldwide. Ratoon cultivation has an important application value and is important for cotton production, breeding, and basic research. In particular, ratooned male-sterile lines have four advantages: an established root system, an indeterminate flowering habit, ratooning ability, and perennial maintenance of sterility in the absence of a matched maintainer. These advantages can help reduce the costs of producing F_1_ hybrid cotton seeds and can help breed high-yielding hybrid combinations because ratooning is a type of asexual reproduction that allows genotypes to remain unchanged. However, ratooning of cotton is highly complex and leads to problems, such as the accumulation of pests and diseases, decreased boll size, stand loss during severe winters, and harmful regrowth during mild winters, which need to be resolved. In summary, ratoon cotton has advantages and disadvantages for the production of hybrid cotton seeds, and future prospects of ratooning annual cotton for the perennial utilization of heterosis are promising if the mechanization of seed production can be widely applied in practice.

## Introduction

### Ratoon Cotton Culture

Cotton is an important cash crop species and the most widely cultivated natural fiber crop species (Ma et al., 2018). China, India, and the United States are currently the three major cotton-planting countries worldwide (Swati and Salunke, 2017). The cotton cultivars currently used in production are mostly annuals. Four different *Gossypium* species are cultivated: *Gossypium herbaceum* and *Gossypium arboreum*, which are diploids (2*n* = 2*x* = 26) that originate from African-Asian stocks, and *Gossypium hirsutum* and *Gossypium barbadense*, which are tetraploids (2*n* = 4*x* = 52) that originate from the Americas; *G. hirsutum* currently contributes more than 90% of the world’s textile fiber (John and Gervers, 2019; Teodoro et al., 2019). The annual cotton cultivars were domesticated from perennial species in tropical and subtropical regions (Huang and Hao, 2018; Jadhav and Bhosale, 2018). However, the annual cultivated species still have perennial habits [i.e., ratooning ability (RA) which mainly refers to the ability of the ratoon crops to maintain cotton yield and fiber quality of the previous growth cycle], which can be utilized for perennial ratooning in tropical or subtropical regions, although the stalks should be pruned before the subsequent season (Zhang et al., 2020). Some studies have shown that the annual species can be ratoon cultivated for approximately 3 years while still producing high yields (Fontes et al., 2006; Zhang et al., 2015a). Cotton has great ratooning potential, as demonstrated by the planting of ratoon cotton as far back as 1786 in Georgia, which represents the earliest known case of ratoon cultivation of cotton worldwide (Seabrook, 1844). Throughout the year in frost-free areas, as soon as the ambient temperature is suitable for growth, the buds of the surviving ratoon cotton begin to sprout; thus, the flowering and fruiting periods of the renewed plants naturally occur approximately 2–6 weeks earlier than do those of the seedlings grown from seeds (Templeton, 1925; Macharia, 2013). Owing to the lack of frost injury during the late growing period and cotton continuing to form new bolls, the yield of renewed cotton plants is greater than that of seed-sown cotton plants. No-tillage cropping of ratoon cotton not only conserves seeds and reduces both labor inputs and soil and water losses but also increases cotton production with limited labor and with ecological benefits (Bergman et al., 1983; Komala et al., 2019). Moreover, some agronomic techniques for use in ratoon cropping of cotton have been proposed, including pruning, pest and disease control, fertilization, and hormone regulation. Notably, among these practices, pruning the stalks is the most important technique to initiate shoot growth; without this step, the ratoon cotton yield would be very low during the following season (Zhang et al., 2020).

### Status and Challenges of Heterosis Utilization in Cotton

Heterosis, also called “hybrid vigor,” is a widespread phenomenon in biology that refers to the superiority of hybrid organisms (heterozygotes) over their parents in one or more traits. For example, hybrids of different strains, varieties, species, and even genera often show a greater growth rate and stronger metabolic function than their parents do. This phenomenon leads to rapid organ development and increases in body size, yield, vitality, fertility, and viability; and in resistance to disease, insects, and stress; etc. (Fu et al., 2014; Ryder et al., 2019). Ever since Joseph Koelreuter, a German scholar, obtained high-yielding, early-maturing, and high-quality tobacco hybrids from hybridization experiments conducted from 1761 to 1766, hybridization has been further developed and utilized for the production of various crop species (Rao et al., 2018; Ryder et al., 2019).

#### Benefits and Promotion of Cotton Hybrids

Cotton hybrids were initially researched in the United States but first applied on a large scale in India and then in China (Sheng et al., 2002). Since Mell (1894) published the first paper on the growth advantages of F_1_ hybrids between upland cotton and sea island cotton, a large number of studies have confirmed that cotton hybrids display obvious interspecific and intraspecific heterosis, i.e., hybrids have stronger advantages than their parents do in terms of seed vigor, yield, fiber quality, and stress resistance. In the 1970s, Mayer developed a cytoplasmic-nucleic (cytoplasmic) male-sterile (CMS) line with *Gossypium harknessii* cytoplasm but failed to use it to breed high-yielding hybrids. In 1970, the world’s first cotton hybrid, “H4,” was released in India and used in production on a large scale (Waghmare, 2016). In 1972, the nucleic (nuclear and genic) male-sterile (NMS/GMS) line “Dong A” was obtained from the cultivar “Dongting No. 1” in China, and more than 10 hybrids were bred in the 1970s (Zhang and Pan, 1999). In addition, researchers in Pakistan, Australia, Israel, the former Soviet Union, and other countries have carried out research on cotton heterosis (Thomson and Luckett, 1988a; Basbag and Gencer, 2010; Soomro et al., 2010).

Hybrid cotton currently occupies up to 96% of the cotton area in India and approximately 80% of the area in the Yangtze River Valley of China (Zhang et al., 2013; Dhillon et al., 2019). Hand-pollination and the use of male-sterile lines are the two major seed production systems for the utilization of heterosis of cotton in China (Zhang et al., 2015a; Wan et al., 2017); the former accounts for approximately 90% of the total F_1_ hybrids, whereas in India, all F_1_ hybrid cotton seeds are produced by hand-emasculation and pollination. The method used in India is feasible, owing to the cheap cost of laborers aged approximately 7–16 years (Galanopoulou-Sendouca and Roupakias, 1999; McKinney, 2014), but unsustainable, as it is illegal to employ children under the age of 14 according to Indian law. The rural labor cost in China is greater than that in India, which is due to the migration of a large number of rural residents to cities (Zhang et al., 2015a; Lin, 2019). Usually, the price of F_1_ hybrid cotton seeds is 100–200% and 35% greater than that of the parental cultivars and F_2_ hybrids, respectively (Dong et al., 2004; Wan et al., 2017). Therefore, F_2_ hybrids, which display less heterosis than do F_1_ hybrids, are widely cultivated in China to reduce the prohibitive cost of cotton seeds compared with that of F_1_ hybrids (Wan et al., 2017).

#### Difficulties and Prospects in Heterosis Utilization of Cotton

Difficulty in breeding high-yielding hybrids and low seed production efficiency are two bottlenecks restricting the sustainable widespread application of cotton heterosis. In particular, F_1_ hybrid cotton seeds are more expensive than other types of cotton seeds because of the greater cost of labor-intensive production associated with the former (Raja et al., 2018). Although cotton can grow perennially in tropical and near-tropical regions, it must be planted in the major cotton-growing regions. Compared with hand-pollination, the use of male-sterile lines to produce hybrid cotton seeds can reduce labor and costs, improve production efficiency, and ensure seed purity (Chen et al., 2005; Wan et al., 2017). Therefore, preliminary analyses have revealed that there may be broad prospects for using ratoon cotton to breed high-yielding hybrid combinations and to produce hybrid seeds with large economic benefits. Further detailed and systematic analysis of the advantages and disadvantages of ratoon cotton is still needed.

## Benefits and Problems Associated with Ratooned Annual Cotton for Heterosis Utilization

Ratooning has been practiced in almost all countries in which cotton is grown (Chamy, 1979). However, in the early twentieth century, this practice was banned by law in some countries to prevent the transfer of pest insects to subsequent crops (Morton, 1975; Mubvekeri et al., 2014). With the development of pest resistance technologies, however, ratoon cotton has been temporarily revived in Peru, South Africa, Zimbabwe, Kenya, and northwestern Australia (Chamy, 1979; Macharia, 2013). Despite the fluctuation in the use of ratoon cotton in production, research on ratoon cotton has been ongoing, and the application of ratoon cotton in breeding has been a popular topic in recent years. Therefore, we have the opportunity to summarize a large number of studies on ratoon cotton to identify consensuses on this topic (Table 1).

**Table 1:** Benefits and problems of using ratoon cotton for heterosis utilization compared to sown cotton.

Class	Ratoon cotton	Reference
Benefits	Original root system	
	Shortened duration	Chamy, 1979; Komala et al., 2019
	Conservation of expensive (hybrid) seeds	Macharia, 2013; Komala et al., 2018c
	Reduced cost of growing a ratoon crop	Macharia, 2013; Komala et al., 2018a
	Fewer weeds because of early canopy closure	Plucknett et al., 1970; Fontes et al., 2006
	Indeterminate flowering habit	
	Extended pollination period	Macharia, 2013; Muhammad et al., 2015
	Increased seed yield	Zhang et al., 2015a; Komala et al., 2018b
	Ratooning ability	
	Observing plant performance yearly	Kumar et al., 2011; Komala et al., 2018c
	Testing field chilling on mature plants	Sachs and Zilkah, 1985; Zhang et al., 2008
	Preserving pathogens for long-term research	Mihail et al., 1987; Seo et al., 2006
	Assessing combining ability	Thomson and Luckett, 1988a; Komala et al., 2019
	Fixing heterosis	Komala et al., 2018d,e
	Maintaining sterility perennially	
	Omitting matched maintainers	Zhang et al., 2013; Zhou, 2016
	Reducing costs associated with producing F_1_ seeds	Zhang et al., 2015a,b
Problems	Multiple ratooning	
	Buildup of pests and diseases	Plucknett et al., 1970; O’Brien, 2016
	Decreased boll size	Macharia, 2013; Zhang et al., 2015a
	Abnormal climate during winter	
	Stand loss during severe winters	Sachs and Zilkah, 1985; Zhang et al., 2008
	Harmful regrowth during mild winter	Greenberg et al., 2007; O’Brien, 2016

Compared with seed-sown cotton, ratooned annual cotton has four major advantages in terms of perennially producing hybrid cotton seeds: (1) preservation of the original root system, which reduces the time needed for root morphogenesis so that the weak seedling stage can be avoided (Chamy, 1979) and the growth duration becomes shortened (Chamy, 1979; Komala et al., 2019), allowing earlier vegetative growth of ratoon cotton when the temperature is suitable and resulting in fewer weeds because of earlier canopy closure (Plucknett et al., 1970; Fontes et al., 2006) and in lower costs associated with cultivation (Komala et al., 2018a); (2) indeterminate flowering habit, which is advantageous by extending the pollination time (Macharia, 2013; Muhammad et al., 2015) and thus results in increased seed yields (Zhang et al., 2015a; Komala et al., 2018b); (3) RA, which offers opportunities for observing annual plant performance (Kumar et al., 2011; Komala et al., 2018c), testing the effects of field chilling on mature plants (Sachs and Zilkah, 1985; Zhang et al., 2008), preserving pathogens over the long term (Mihail et al., 1987; Seo et al., 2006), and assessing combining ability and heterosis (Thomson and Luckett, 1988a,b; Komala et al., 2018b, 2019); and (4) the perennial maintenance of male sterility in the absence of a matched maintainer (Zhang et al., 2013; Zhou, 2016), which reduces the cost of F_1_ seeds (Zhang et al., 2015a,b). Therefore, the use of ratooned male-sterile lines to produce low-cost hybrid cotton seeds has good application prospects.

However, there can be problems associated with ratooned annual cotton, such as: (1) multiple ratooning, which facilitates the accumulation of overwintering pests and diseases (Plucknett et al., 1970; O’Brien, 2016) and leads to reductions in boll size (Macharia, 2013; Zhang et al., 2015a), and (2) susceptibility to an abnormal climate during winter, which has two different effects – stand loss during severe winters (Sachs and Zilkah, 1985; Zhang et al., 2008) and harmful regrowth during mild winters (Greenberg et al., 2007; O’Brien, 2016). In addition, the dramatic increase in labor costs requires implementation of a mechanized one-off cotton harvest, which is difficult to achieve in ratooned annual cotton with a long boll-opening period (Zhang et al., 2020).

## Evaluation of the RA of Cotton

The calculation of the RA of cotton is in reference to the calculation of the RA of ratoon crops (RCs) of sugarcane, which is expressed as a percentage and is obtained by dividing the value of the RC with that of the planted crop (PC) for the same trait “i”; i.e., RA_i_ = RC_i_/PC_i_ (Komala et al., 2018f,g). The RA of the parents determines the yield of the hybrid seeds, and the RA of the hybrids indicates their ability to maintain their own heterosis.

In addition to the agronomic and environmental factors considered roughly the same in a study, the RA of cotton is also affected by the genotype, crop age, and their interactions. For all the traits of ratoon cotton studied by Komala et al. (2018d), there were significant differences in RA among the genotypes evaluated, and similar phenomena were reported for ratoon sugarcane (Ogunniyan et al., 2018; Chumphu et al., 2019). Within the same cotton genotype, there were significant differences in boll number per plant, boll weight, lint index, and seed cotton yield per plant between the PCs and the RCs (Komala et al., 2018d). In addition, the yield of ratoon cotton and its contributing traits are affected by crop age. The interaction between genotype and crop age significantly affected all the traits studied, which indicates that hybrid performance differs during different cropping cycles. Studies have shown that the interaction between genotype and crop age is highly important to yield its contributing traits (Ahmed et al., 2016; Komala et al., 2018d; Chumphu et al., 2019). Therefore, RA_j_ = RC_j_/PC can be used to calculate the RA of crops at different ages (j). If the effects of a trait (i) and crop age (j) are considered simultaneously, RA_ij_ = RC_ij_/PC_i_ can be used to calculate the RA. According to different trials, the RA of seed cotton ranged from 63.81 to 218.67% (Table 2).

**Table 2:** Ratooning ability of seed-cotton yield in different trials.

Material	Cropping	RA	Location	Year	Reference
*Gossypium hirsutum* cv. AC 134	Perennial	RA_2_ = 175.44%	Lyallpur, Pakistan	1971–1972	Khan and Shabbir, 1974
*G. hirsutum* cv. Xiangzamian 3 F_2_	Perennial	RA_2_ = 124.14% RA_3_ = 106.80%	Nanning, China	2005–2007	Chen et al., 2008, 2010
*G. hirsutum* GMS line Dong A	Perennial	RA_2_ = 218.67% RA_3_ = 150.79%	Nanning, China	2005–2007	Zhang et al., 2010
*G. hirsutum* cv. Chuanzamian 15 F_1_	Perennial	RA_2_ = 110.28% RA_3_ = 105.87% RA_4_ = 97.577% RA_5_ = 90.65% RA_6_ = 84.06%	Panzhihua and Lijiang, China	2006–2012	Zhang et al., 2015a,b
*G. hirsutum* cv. HART 89 M	Perennial	RA_2_ = 83.11%	Kirinyaga, Kenya	2007–2009	Macharia, 2013
*G. hirsutum* cv. Suraj	Biannual	RA_2_ = 63.81%	Coimbatore, India	2012–2013	Khader and Prakash, 2014
*G. barbadense* cv. Suvin	Biannual	RA_2_ = 64.10%	Coimbatore, India	2012–2013	Khader and Prakash, 2014
*G. hirsutum* TCH 1716 × Suvin	Perennial	RA_2_ = 99.42%	Coimbatore, India	2016–2017	Komala et al., 2018f
*G. hirsutum* VS 9-S11-1	Perennial	RA_2_ = 98.31%	Coimbatore, India	2016–2017	Komala et al., 2018d

RA, ratooning ability; AC, American cotton; GMS, genic male sterile.

## Use Of Ratoon Cotton for the Production of Commercial F_1_ Hybrid Cotton Seeds

At present, the development of hybrids is the key to increasing cotton yield, and ratoon cotton can produce maximum seed yields under conditions of early plant maturity, which makes it possible to reduce the costs of hybrid cotton seeds for commercial production. Ratooned parents with high seed yield have the potential to produce efficient hybrid cotton, while ratooned hybrids with high lint yield can be used in commercial cotton production. In terms of seed cotton yield per plant, the positive standard heterosis of intra-*hirsutum* hybrids was determined to reach 68.86% (Komala et al., 2018d). Therefore, utilizing ratoon cotton to produce inexpensive F_1_ hybrid cotton seeds with high heterosis would be an effective way to improve cotton yield and quality rapidly (Zhang et al., 2020).

### Use of Ratoon Cotton in the Production of F_1_ Hybrid Cotton Seeds by Hand-Emasculation

Currently, the production of hybrid cotton seeds still occurs predominantly by the traditional method of hand-emasculation and pollination (Gupta et al., 2012). The main advantage of hand-emasculation is that it allows easy obtainment of strong, dominant combinations through free matching. Cotton flowers are large, which is conducive to hand-emasculation and pollination, and during the flowering period, a skilled worker can pollinate hundreds of flowers a day. Widely used cotton hybrids in India (Galanopoulou-Sendouca and Roupakias, 1999) and China (Wan et al., 2017) were obtained in this way. However, the hand emasculation process is labor-intensive, which increases the cost of hybrid seed production (Zhang et al., 2015b).

### Ratooning Male-Sterile Cotton for the Production of F_1_ Hybrid Cotton Seeds

Hand-emasculation can easily damage ovaries, and the resulting seed setting rate ranges from 7 to 35%. Especially in the case of interspecies hybrids, this makes hybrid seeds expensive and hinders the utilization of cotton heterosis to a certain extent (Gopal, 2018). Conversely, improved hybrid seeds developed by the use of male-sterile lines can achieve greater seed set rates because mechanical damage to the ovary can be avoided during hand-emasculation, thereby reducing the cost of producing hybrid seeds by considerably decreasing the labor requirements and improving efficiency (Chen et al., 2005; Wan et al., 2017).

There are two types of male sterility of cotton, CMS and NMS. Notably, it is more difficult to find combinations with strong heterosis *via* the CMS 3-line method than *via* the NMS 2-line method.

#### Maintenance of Male Sterility of Sown Cotton by Ratooning

Researchers are currently exploring new strategies to utilize heterosis in cotton. In China, the NMS line “Dong A” has the advantages of being stably male sterile, and its fertility is easily restored; thus, it has been extensively used for producing hybrid seeds in Sichuan, China (Zhang et al., 2013). When NMS lines are used as female parents in cotton seed production, 50% of the fertile plants in the first year are removed at the flowering stage, and the remaining 50% of the sterile plants are retained for seed production. When the latter mature, the hybrid seeds are harvested and then used the following year. All plants become sterile during the next year, which is beneficial because it reduces seed production costs, improves hybrid purity, and allows insects to supplement pollination (Zhang et al., 2015a).

With respect to the ratoon plants, when the fertile restorer:NMS ratio ranged from 1:2 to 1:4, the cost of producing hybrid F_1_ cotton seeds decreased by 18% compared with that of the two-line method without ratooning and by 60% compared with that of the hand-pollination method. When this method is applied specifically in the tropics and near-tropics, many wild pollinating insects are present, which could further reduce the amount of labor needed for hand-pollination by 16% (Zhang et al., 2015a). Additionally, vegetative propagation, including cutting and grafting, can be used to generate NMS plants, which could omit the need to identify and remove fertile plants.

#### Maintenance of the Male Sterility of Cutting-Propagated Plants by Ratooning

The fertility of NMS lines is easy to restore but difficult to maintain by sexual reproduction. Thus, fertile plants, which account for approximately 50% of the NMS lines, should be removed on the basis of the fertility of the flowers on each plant during the flowering period (Zhang et al., 2015a). This work involves a great deal of labor and decreases the F_1_ cottonseed yield. Even the two-step propagation method of NMS lines for producing hybrid cotton seeds is complex, and maintaining the NMS line is difficult. Thus, the maintenance of the male sterility of cutting-propagated plants by ratooning may be a good way to resolve the above problems.

Ratoon cropping for propagating NMS cotton by cuttings to produce hybrid seeds was shown to be feasible in Nanning, China. There was no difference in terms of yield or fiber quality between the F_1_ hybrids of the male-sterile line “Dong A” with or without ratoons and the same male parent (Zhang and Zhou, 2009). This method does not require land preparation or the sowing of seeds each year, which can reduce the input of raw materials needed for production and labor. Furthermore, less rouging and sister crossing would be needed, which could simplify the procedures of producing hybrid cotton seeds and reduce the costs associated with planting NMS lines, and the yield and purity of the hybrid cotton seeds could be improved. However, cutting-propagated plants cannot overwinter safely when the winter temperature is below normal (as was the case in 2008; Zhang et al., 2008; Zhang and Zhou, 2009).

#### Maintenance of Male Sterility of Grafted Plants by Ratooning

**Figure 1:**
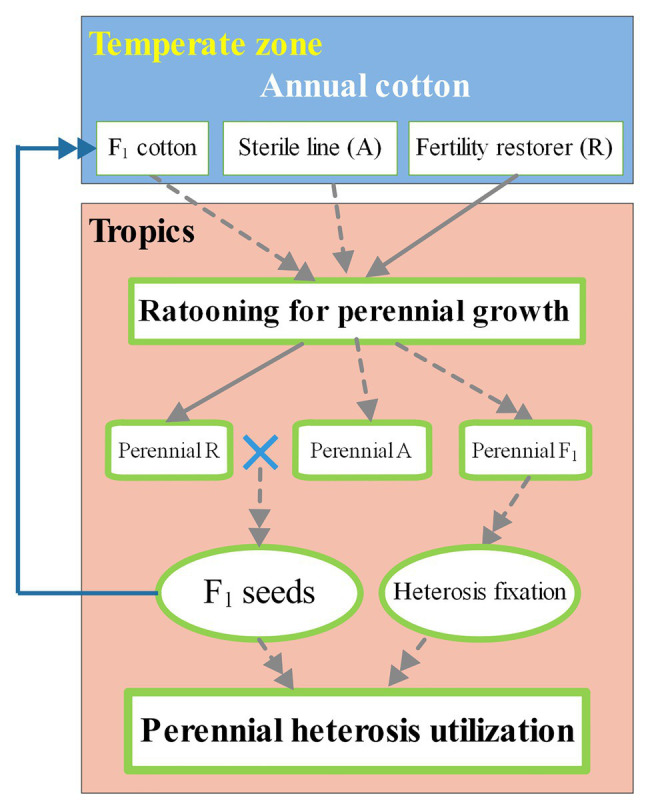
Ratooning annual cotton for perennial heterosis utilization in tropical and near-tropical areas. The perennial heterosis utilization (green-bordered box with bold font) of annual cotton (white box) includes the production of hybrid cotton seeds generated by a male-sterile line (dotted line with a single arrowhead) and a male fertility restorer (solid line with a single arrowhead), followed by heterosis fixation of hybrid cotton (dotted line guided by double arrowheads). In subtropical regions (orange box), annual cotton from the temperate zone (blue box) can be grafted onto perennial cotton with strong cold resistance to improve winter survival. All perennial cottons types are shown in green-bordered boxes.

In the near-tropics, annual cotton cultivars can be used as scions for grafting onto rootstocks of perennial species to achieve long lifespans with high and stable yields (Zhang et al., 2012a,b). A new method of propagating and cultivating NMS cotton for producing hybrid seeds perennially by grafting has been proposed (Zhang et al., 2013; Zhou, 2016). Likewise, this method could be used for CMS cotton. Moreover, annual cotton cultivated with this method could grow over more cycles than normal ratoon cotton because of the use of perennial species as rootstocks. The utilization of heterosis of annual cotton for many years in near-tropical areas is illustrated in Figure 1.

This method offers the greatest economic potential for planting ratoon cotton in the tropics and near-tropics (Zhang et al., 2013, 2015a). Therefore, breeding male-sterile cotton lines with strong overwintering survival and good comprehensive traits and utilizing the advantages of ratoon cropping to maintain their sterility to produce hybrid seeds could reduce the current costs associated with hybrid seed production (Zhou, 2016). Moreover, notably, in the case of the large-scale production of hybrid cotton seeds, bees can be artificially bred for use in improving the purity of hybrid seeds (Zhang et al., 2015a). Additionally, high temperatures can cause problems, such as traces of fertile pollen occurring in male-sterile cotton in the tropics, which can be resolved by increasing heat resistance during breeding.

## Future Studies on Ratoon Cotton for Heterosis Utilization

Although there are many advantages associated with ratooning annual cotton for heterosis utilization, insufficient research has been conducted in this field. It is critical to study the control of pests, overwintering management, and breeding techniques for ratooning annual cotton for heterosis utilization, the results of which will provide necessary and economical means to achieve maximum yields and benefits.

### Development of Integrated Pest Management for Ratooning Annual Cotton

A large number of reports have shown that pests are not a major problem in ratoon cotton and that the impact of pests on RCs is even lower than that on PCs (Templeton, 1925; Evenson, 1970; Khader and Prakash, 2014). However, there is abundant evidence that failure to control pests effectively can severely impact the yield of ratoon cotton (Plucknett et al., 1970; Sampaio et al., 2017). Compared with sown cotton, ratoon cotton experiences earlier attacks by aphids, bollworms, and mealybugs. In addition, Vitale et al. (2007) reported that even if the plants were sprayed at approximately six times the recommended rate per season for *Bacillus thuringiensis* (Bt) cotton, approximately 23% of the seed cotton yield would still be lost due to pests. Since the 1990s, lepidopteran pests, such as bollworms, have been effectively controlled because of the large-scale cultivation of Bt cotton, but the occurrence of sucking pests, such as aphids, leaf mites, mirids, and mealybugs, has been increasing (Ghelani et al., 2014; Nikam, 2017; Huang and Hao, 2018). Moreover, the overuse of agrochemicals has resulted in a large number of deaths of natural enemies and has accelerated the increase in cotton pest resistance. The resistance of cotton aphids to pyrethroids has increased by 1,000-fold, and the resistance to imidacloprid has increased more than 500-fold. In addition, the resistance of bollworms to pyrethroid pesticides is still high, and their resistance to Bt cotton is increasing each year. Therefore, it is difficult to control pests in ratoon cotton economically and effectively by one or two methods, and integrated pest management (IPM) must be adopted (Zhang et al., 2020).

In 1967, Smith and Van-den Bosh first proposed the term “integrated pest population management,” and IPM was formally accepted by the scientific community in 1972 (Ha, 2014). IPM has essentially become a basic agroecological method for pest management, starting with classic biological control and host-plant resistance, and includes recent targeting methods for plant species diversification, such as push-pull techniques (Praharaj et al., 2010), pesticides (such as chitin synthesis inhibitors) that are lethal to insects but less toxic to vertebrates (Reda et al., 2010), and landscape management methods to enhance biological control (Downes et al., 2017). IPM currently includes the use of insect-resistant and early-maturing cotton varieties, various cultivation methods, pest resistance management, the use of economic thresholds through reconnaissance, and the timely application of pesticides when needed. In practical applications, both the effectiveness and economic factors should be considered. For example, the assassin bugs *Pristhesancus plagipennis* and *Trichogramma chilonis* can be used for the biological control of bollworms, but compared with the use of Bt cotton and pesticides, the use of these assassin bugs is still uncommon because of the greater costs associated with their release (Sahayaraj et al., 2012; Khan, 2019).

Among all the IPM methods, the use of insect-resistant cotton is the most economical and effective. In traditional insect-resistant cotton breeding, the use of cotton morphological mutants to control pest reproduction may be possible. For example, Silva et al. (2008) investigated the behavior of the boll weevil (*Anthonomus grandis*) when attacking ratooned upland cotton mutants whose morphological characteristics included okra-shaped leaves, frego bracts, and red coloration, and the results clearly showed that the number of eggs laid per red plant with frego bracts was lower than that laid on the other plant types. In addition to the use of transgenic methods to breed insect-resistant cotton, emerging molecular biology technologies, such as gene editing and RNA-interference (RNAi) gene silencing technologies, are being used (Bolaños-Villegas, 2020).

### Overwintering Management of Ratooned Annual Cotton

During winter in the tropics, cotton plants are pruned after harvest, and some regenerated cotton can even blossom and bear fruit; thus, the overwintering of ratoon cotton is not a problem. However, low temperatures are prevalent in winter in subtropical regions, which limits cotton overwintering and RA. Chilling damage in the field is obviously determined by many factors, including various climatic conditions, such as the duration of low temperature and rainfall, soil conditions, physiological conditions of the plants, cultivation methods, and plant species (Zhang et al., 2020).

During the winter in Israel, Sachs and Zilkah (1985) attempted to alleviate damage to cotton plants caused by low temperatures in the field. The height of the cotyledons was reached or exceeded by piling soil onto the stems of the plants, which improved local drainage and increased the survival rate of the plants by 13%. Furthermore, increasing the ambient temperature by covering the rows of plants with polyethylene sheets could reduce injury by more than 20%, but this method is neither economical nor environmentally friendly. Throughout the winter, it was determined that leaving the cotton plants unpruned was better than all the other pruning methods. In addition, applications of fertilizer, plant growth regulators, straw mulching, grafting, and other methods can improve the survival rate of cotton plants during the winter (Zhang et al., 2020). However, the most economical method is to breed varieties that present high yield and fiber quality, pest resistance, and strong overwintering ability (Zhang et al., 2013).

During winter in subtropical regions, it is best to allow cotton plants used for ratooning to enter dormancy. However, under warm winter conditions, sprouts can form on cotton plants, which consume accumulated assimilates and become food for overwintering pests (Zhang et al., 2020). Therefore, after cotton harvest, it is best to spray plant growth regulators on cotton plants to facilitate dormancy for winter survival.

### Ratooning Annual Cotton to Breed Hybrid Combinations That Display High Heterosis

Ratooned annual cotton can be used to breed strong hybrid combinations due to a static genotype and is used to produce hybrid seeds owing to the increased yield and constant fiber quality in the first and even second generation of RCs.

The conventional technique of hybrid breeding involves analyzing the heritability of important traits such as yield, fiber quality, maturity, and resistance in the parent, F_1_, and subsequent generations (Komala et al., 2018a,d). For traits with high narrow-sense heritability, selective breeding should be adopted, and if broad-sense heritability is high, cross-breeding is generally considered appropriate. On the other hand, if one trait is controlled mainly by additive genes, simple selection procedures, such as pedigree breeding, are sufficient (Komala et al., 2018g). However, most traits have a dominant gene effect, which indicates that selection should be postponed to the offspring after crossing. Cross-breeding is an effective way to make full use of dominant genes (Komala et al., 2018g). In cross-breeding, the general combining ability and the specific combining ability of a ratoon trait can be identified through the analysis of line × tester crosses (Komala et al., 2018g). The general combining ability reflects the additive effect, while the specific combining ability involves the non-additive effect (Komala et al., 2018b). Therefore, a strategy for breeding hybrid cotton combinations that display strong heterosis could be as follows: “The parents should be varieties with good comprehensive characteristics, no obvious defects, complementary advantages, and a high general combining ability.”

With the advancement of modern molecular biology, it will be increasingly effective to use genetic markers, quantitative trait loci (QTLs), and genome-wide association studies (GWASs) for selecting parents that present high yields, good quality, early maturity, and excellent resistance for hybrid combinations (Xia et al., 2014; Diouf et al., 2018; He et al., 2018; Thyssen et al., 2019).

### Problems Associated With the Mechanization of Ratoon Cotton for Seed Production

Cotton is a more labor-intensive field crops species than wheat, corn, and soybean (Nazir et al., 2018). In the current context of accelerating urbanization worldwide, the shortage of the labor force in rural regions is becoming an increasingly prominent factor affecting agricultural production, so the demand for complete mechanization of cotton production is increasing in urgency.

The complete mechanization of cotton production involves land preparation, seed sowing or seedling transplanting, plant protection, middle plowing and topdressing, harvest, cotton stalk pulling, and depilation, among which the mechanization of harvest is currently the weakest factor and is also an important bottleneck restricting the scale of ratooning annual cotton for seed production. However, because the scale of cottonseed production is much smaller than that of raw cotton production and because a large-scale cotton picker is a large one-time investment, during the initial stage of cotton seed production by ratooning, some small machines, including portable hand-held cotton pickers, can be used to collect seed cotton (Mohanty and Deshmukh, 2016; Majumdar et al., 2019). In some tropical rural areas where the price of labor is relatively cheap, compared with machine-picked cotton, hand-picked cotton not only is less expensive but also results in better fiber quality (Tian et al., 2018).

Of particular importance, considering that it is difficult to manage and harvest perennial cotton plants mechanically because of their uneven growth, it is necessary to adopt a biannual cropping system of annual cotton (i.e., an RC followed by a PC within 1 year) to obtain yields greater than those of perennial cotton and to avoid the accumulation of overwintering pests caused by the latter (Zhang et al., 2020). In addition, the mechanization of ratoon cotton for seed production also involves issues such as cotton breeding, planting patterns, accelerated boll maturation and defoliation, cotton seed processing, and quality standards.

## Conclusion

Ratoon cropping is highly important to cotton production, the permanent maintenance of the male-sterile line for heterosis utilization, the fixation of heterosis, and the preservation and generation of novel germplasm. Therefore, taking advantage of the warm climate to exploit the perennial and indeterminant growth habits of cotton would be profitable, resource-saving, and environment-friendly in the tropics. However, increased investments are needed for ratoon cotton breeding, cropping, and agro-ecological research.

Although ratooning of annual cotton for heterosis breeding has been successful in some countries, such as Australia, China, and India, owing to the lack of results from evaluations of commercial production and sales of hybrid cotton seeds from ratooning systems, we can only hypothesize that this practice has good future prospects. In addition, the expansion of ratoon cotton would not necessitate the use of more natural and seminatural land for agricultural development because if tropical ratoon cotton is used for lint production, its economic benefit is much lower than that of hybrid seed production or even lower than that of temperate annual cotton, and the management of ratoon cotton used to produce hybrid seeds necessitates more labor than that needed for grain crops.

## Author Contributions

XZ, ZZ, and LW conceived and designed the contents of the manuscript. RZ and QW analyzed the available literature. XZ wrote the paper. All authors contributed to the article and approved the submitted version.

### Conflict of Interest

The authors declare that the research was conducted in the absence of any commercial or financial relationships that could be construed as a potential conflict of interest.
